# One-field, two-field and five-field handheld retinal imaging compared with standard seven-field Early Treatment Diabetic Retinopathy Study photography for diabetic retinopathy screening

**DOI:** 10.1136/bjo-2022-321849

**Published:** 2023-04-24

**Authors:** Recivall P Salongcay, Cris Martin P Jacoba, Claude Michael G Salva, Abdulrahman Rageh, Lizzie Anne C Aquino, Aileen V Saunar, Glenn P Alog, Mohamed Ashraf, Tunde Peto, Paolo S Silva

**Affiliations:** 1 Centre for Public Health, Queen's University Belfast, Belfast, UK; 2 Philippine Eye Research Institute, University of the Philippines Manila, Manila, Philippines; 3 Eye and Vision Institute, The Medical City, Pasig City, Philippines; 4 Joslin Diabetes Center, Beetham Eye Institute, Department of Ophthalmology, Harvard Medical School, Boston, Massachusetts, USA; 5 Faculty of Medicine, Alexandria University, Alexandria, Egypt

**Keywords:** Imaging, Retina, Public health, Telemedicine

## Abstract

**Background/aims:**

To determine agreement of one-field (1F, macula-centred), two-field (2F, disc–macula) and five-field (5F, macula, disc, superior, inferior and nasal) mydriatic handheld retinal imaging protocols for the assessment of diabetic retinopathy (DR) as compared with standard seven-field Early Treatment Diabetic Retinopathy Study (ETDRS) photography.

**Methods:**

Prospective, comparative instrument validation study. Mydriatic retinal images were taken using three handheld retinal cameras: Aurora (AU; 50° field of view (FOV), 5F), Smartscope (SS; 40° FOV, 5F), and RetinaVue (RV; 60° FOV, 2F) followed by ETDRS photography. Images were evaluated at a centralised reading centre using the international DR classification. Each field protocol (1F, 2F and 5F) was graded independently by masked graders. Weighted kappa (Kw) statistics assessed agreement for DR. Sensitivity (SN) and specificity (SP) for referable diabetic retinopathy (refDR; moderate non-proliferative diabetic retinopathy (NPDR) or worse, or ungradable images) were calculated.

**Results:**

Images from 225 eyes of 116 patients with diabetes were evaluated. Severity by ETDRS photography: no DR, 33.3%; mild NPDR, 20.4%; moderate, 14.2%; severe, 11.6%; proliferative, 20.4%. Ungradable rate for DR: ETDRS, 0%; AU: 1F 2.23%, 2F 1.79%, 5F 0%; SS: 1F 7.6%, 2F 4.0%, 5F 3.6%; RV: 1F 6.7%, 2F 5.8%. Agreement rates of DR grading between handheld retinal imaging and ETDRS photography were (Kw, SN/SP refDR) AU: 1F 0.54, 0.72/0.92; 2F 0.59, 0.74/0.92; 5F 0.75, 0.86/0.97; SS: 1F 0.51, 0.72/0.92; 2F 0.60, 0.75/0.92; 5F 0.73, 0.88/0.92; RV: 1F 0.77, 0.91/0.95; 2F 0.75, 0.87/0.95.

**Conclusion:**

When using handheld devices, the addition of peripheral fields decreased the ungradable rate and increased SN and SP for refDR. These data suggest the benefit of additional peripheral fields in DR screening programmes that use handheld retinal imaging.

**WHAT IS ALREADY KNOWN ON THIS TOPIC**

- Majority of diabetic retinopathy (DR) screening programmes using conventional tabletop retinal cameras use one or two field imaging protocols. However, studies on tabletop cameras have shown that the inclusion of peripheral fields may improve the accuracy of retinal imaging.

**WHAT THIS STUDY ADDS**

- Given that handheld retinal devices typically have higher ungradable rates and lower agreement rates compared with tabletop cameras, the addition of peripheral fields when using handheld retinal imaging devices decreased ungradable rate and increased the identification of referable DR.

**HOW THIS RESEARCH MIGHT AFFECT RESEARCH, PRACTICE OR POLICY**

- These data suggest a potential benefit of the addition of peripheral fields to improve detection of referable disease in DR screening programmes that use handheld retinal imaging.

## Introduction

As the number of people with diabetes continues to increase worldwide, retinal imaging and teleophthalmology for diabetic retinopathy (DR) screening are becoming increasingly important as a clinical exam with eye care providers is not always accessible, especially in rural areas and low-income to middle-income countries (LMICs).^1 2^ The introduction of innovative digital technology by handheld retinal imaging devices seeks to augment this area of need. Handheld retinal imaging devices are highly portable and relatively inexpensive equipment that can significantly expand DR screening initiatives, allowing a much larger population to be evaluated. These compact cameras may be more widely deployed in underserved areas and can be used by allied health personnel, primary care physicians and non-medical staff with proper training.^3–5^ Hence, it is essential that these devices accurately identify the DR severity in patients’ eyes if diabetic retinopathy screening programmes (DRSPs) were to rely on these instruments.

Finalising the protocol on using these handheld devices involves outlining the recommended photographic technique for capturing fundus images on patients to maximise their sensitivity (SN) and specificity (SP). At least one macula-centred image is standard of care in published studies. However, there are different views on using additional fields for screening purposes.^6–8^ Moreover, most studies comparing the effect of field number on DR screening accuracy investigated tabletop cameras.^7–9^ Handheld cameras differ significantly from tabletop devices but offer a potentially more cost-effective alternative in rural areas and LMICs, where screening initiatives are needed the most due to the higher burden of vision loss from DR in these locations.^10^

The purpose of the present study is to evaluate the effect of the number of retinal fields on mydriatic handheld retinal imaging protocols for the assessment of DR compared with standard Early Treatment Diabetic Retinopathy Study (ETDRS) seven-field stereoscopic 30° fundus photographs (ETDRS photos).

## Materials and methods

### Population and sample

This was a single-site, cross-sectional, comparative instrument validation study for the detection and evaluation of DR. A total of 225 eyes from 116 patients with diabetes were recruited from The Medical City, Metro Manila, Philippines. Fundus photos taken during the same visit were prospectively collected from the study participants.

Inclusion criteria were (1) 18 years of age or older at the time of informed consent, (2) diagnosis of type 1 or 2 diabetes mellitus (DM) and (3) willingness to undergo mydriatic retinal imaging procedures. Exclusion criteria were (1) media opacity that prevents adequate view of the retina, (2) history of hypersensitivity to mydriatic eye drops or any evidence of contraindication to pupil dilation, and (3) eyes with active ocular infection or inflammation at the time of examination.

### Imaging and grading protocol

Study participants underwent pupil dilation using one drop of tropicamide 0.5%+phenylephrine 0.5% eye drops. Mydriatic fundus photos were acquired using three handheld retinal cameras: (1) Aurora (AU; Optomed, Oulu, Finland), (2) Smartscope (SS, Optomed) and (3) RetinaVue (RV) 700 (Welch Allyn, Skaneateles Falls, New York, USA), and stereoscopic 30° standard seven-field ETDRS fundus photos were acquired using a tabletop retinal camera (VISUCAM; Carl Zeiss Meditec, Dublin, California, USA).

**Figure 1:**
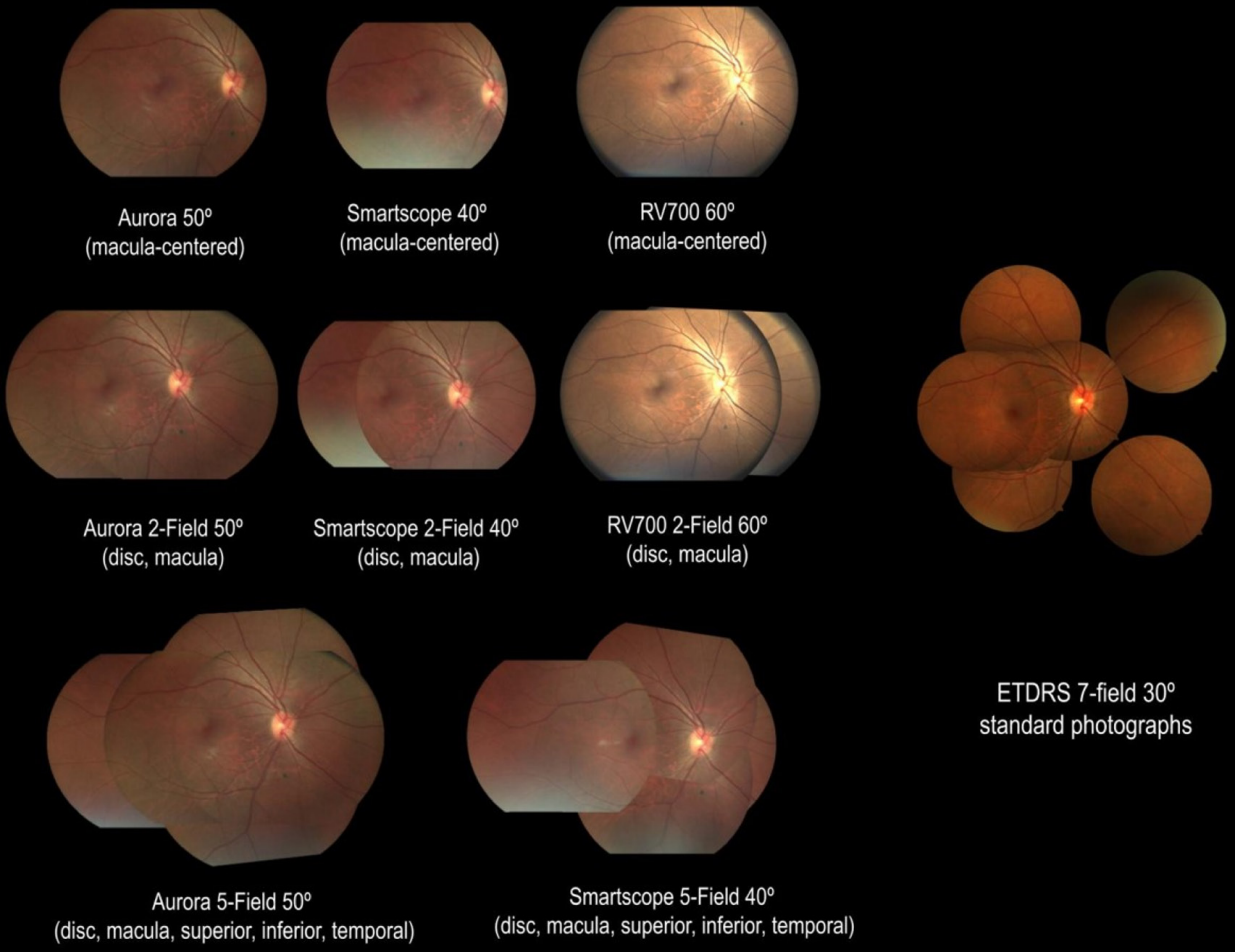
Montage of one-field (A), two-field (B) and five-field (C) imaging protocols on the Aurora handheld device compared with standard seven-field ETDRS photos (D). ETDRS, Early Treatment Diabetic Retinopathy Study.

**Figure 2:**
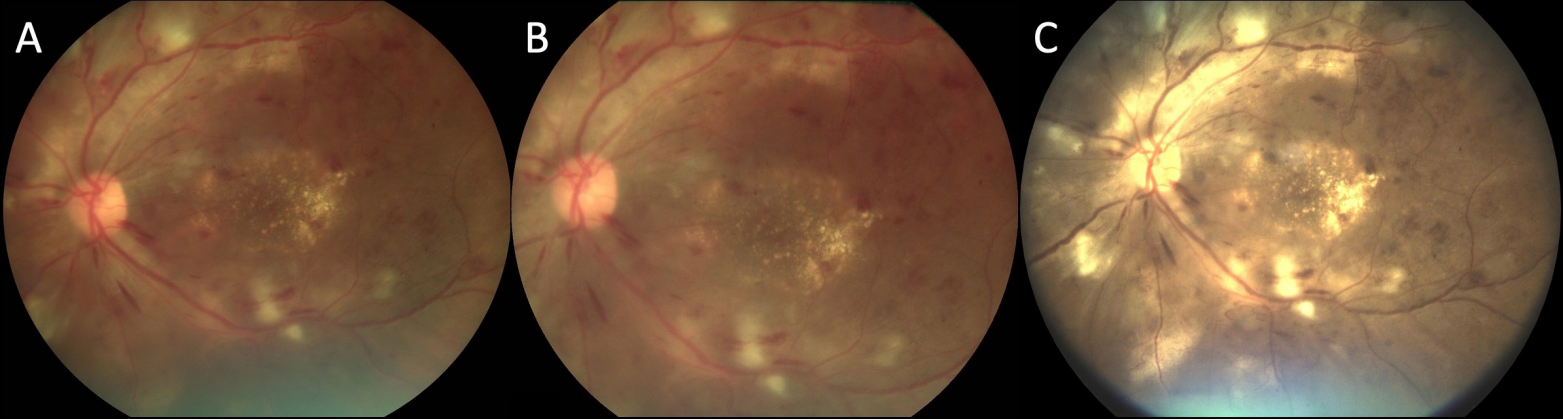
Montage of macula-centred images taken with Smartscope (A), Aurora (B) and RetinaVue 700 (C) handheld retinal imaging devices. The patient was graded as having proliferative diabetic retinopathy on seven-field ETDRS photographs. Retinal neovascularisation elsewhere is seen most prominently along the distal superotemporal arcade. ETDRS, Early Treatment Diabetic Retinopathy Study.

Forty-degree one-field (1F, macula centred), two-field (2F, macula-centred and disc-centred) and five-field (5F; disc-centred, macula-centred, superior periphery, inferior periphery and temporal periphery) fundus photos were acquired using SS, along with 50° 1F, 2F and 5F fundus photos using AU and 60° 1F and 2F fundus photos using RV. 5F photos were not taken using RV due to the manufacturer’s device configuration limitations. The order of imaging using the handheld retinal cameras was based on device availability and was mostly random. Following handheld retinal imaging, 30° seven-field stereoscopic fundus photos were acquired. Figure 1 presents a comparison of the 1F, 2F and 5F retinal images taken using handheld cameras and the standard seven-field ETDRS photos. Figure 2 compares left eye images with proliferative diabetic retinopathy (PDR) taken with the three different handheld cameras. All handheld retinal images were acquired by trained imager–graders certified by the Gloucestershire Retinal Education Group (Gloucestershire Hospitals NHS Foundation Trust, UK), and ETDRS photos were acquired by clinical trial certified ophthalmic photographers. All collected images were deidentified and stored in a secure location.

All study images were evaluated independently by five trained graders (two certified image graders (LACA and CMGS), two board-certified ophthalmologists (GPA and AVS) and one retina specialist (RPS)) who were masked to the diagnosis in either eye. Image grading was performed at a centralised reading centre using high-resolution, high-definition LCD computer displays. These display monitors were regularly colour-calibrated to a colour temperature of 6500 K and gamma setting of 2.2 (Spyder4PRO; Datacolor, Lawrenceville, New Jersey, USA). DR was graded using the international clinical DR classification (no DR, mild non-proliferative diabetic retinopathy (NPDR), moderate NPDR, severe NPDR, PDR or ungradable). A senior retina specialist (PSS) adjudicated disagreements among the graders.

The digital image time stamps were reviewed to provide insight on acquisition time.

### Statistical analysis

Sample size calculation was determined using kappa values projected to be 0.7–0.8. With a power of 80%, the sample size is 206 eyes.^11^ Adjusting for an estimated 10% ungradable rate, 225 eyes were enrolled. Simple kappa (K) and weighted kappa (Kw) statistics were measured to assess the level of agreement for DR between the 1F, 2F and 5F images taken using handheld retinal cameras and ETDRS standard seven-field photos. Kappa values of 0.41–0.60 were classified as having moderate agreement, 0.61–0.80 as substantial agreement, and 0.81 or higher as near-perfect agreement. SN and SP values were calculated for any DR, referable diabetic retinopathy (refDR; moderate NPDR or worse or ungradable images) and vision-threatening diabetic retinopathy (vtDR; severe NPDR or worse or ungradable images) and were compared against established SN and SP thresholds of 0.80 and 0.95, respectively.^12^ SAS software V.9.4 was used for statistical analysis.

## Results

One hundred sixteen study participants (225 eyes) were included. The mean age of study participants was 58.6 (SD ±10.5); 48 out of 116 were men (41.4%) and 68 (58.6%) were women. Using standard seven-field ETDRS photos, results showed that 75 (33.3%) eyes had no DR; 46 (20.4%) had mild NPDR; 32 (14.2%) had moderate NPDR; 26 (11.6%) had severe NPDR; and 46 (20.4%) had PDR. No eye was ungradable for DR on ETDRS photos. Table 1 summarises the baseline characteristics of patients and DR severity by ETDRS photos.

**Table 1:** Baseline characteristics and DR severity by ETDRS photos

	Value±SD or N (%)
Characteristics	
Female sex	68 (58.6)
Age (years)	56.8±10.5
Average A1c	7.3±1.6
Hypertension	65 (56.0)
Renal disease	12 (10.3)
DR severity by ETDRS photos	
No DR	75 (33.3)
Mild NPDR	46 (20.4)
Moderate NPDR	32 (14.2)
Severe NPDR	26 (11.6)
PDR	46 (20.4)
Ungradable	0

DR, diabetic retinopathy; ETDRS, Early Treatment Diabetic Retinopathy Study; NPDR, non-proliferative diabetic retinopathy; PDR, proliferative diabetic retinopathy.

All handheld cameras achieved moderate to substantial agreement levels for DR severity when compared with ETDRS photos. For 1F photos, RV had the highest agreement (Kw=0.77; exact 59.6%, within one step 90.7%) with ETDRS photos compared with AU (Kw=0.54; 44.4%, 81.3%) and SS (Kw=0.51; 42.2%, 75.6%). Similarly, RV had the highest agreement (Kw=0.75; exact 63.1%, within one step 88.9%) with ETDRS for 2F photos, higher than both AU (Kw=0.59, 48.4% and 85.8%) and SS (Kw=0.60, 49.3% and 84.0%). AU had a higher agreement (Kw=0.75; exact 65.8%, within one step 93.8%) with ETDRS photos than SS (Kw=0.73; 60.0% and 90.7%) for 5F photos. Looking at the results per device, the agreement with ETDRS photos increased with increasing number of fields for AU (1F: Kw=0.54, exact 44.4%, within one step 81.3%; 2F: 0.59, 48.4%, 85.8%; 5F: 0.75, 65.8%, 93.8%). This trend is also seen with SS (1F: 0.51, 42.2%, 75.6%; 2F: 0.60, 49.3%, 84.0%; 5F: 0.73, 60.0%, 90.7%) but not for RV (1F: 0.77, 59.6%, 90.7%; 2F: 0.75, 63.1%, 88.9%).

SN/SP for any DR, refDR and vtDR were 1F-AU 0.69/0.99, 0.72/0.92, 0.80/0.91; 2F-AU 0.72/0.99, 0.74/0.92, 0.81/0.89; 5F-AU 0.88/0.97, 0.86/0.97, 0.84/0.92; 1F-SS 0.72/0.85, 0.72/0.92, 0.67/0.92; 2F-SS 0.76/0.82, 0.75/0.92, 0.75/0.91; 5F-SS 0.83/0.92, 0.88/0.92, 0.87/0.86; 1F-RV 0.78/0.93, 0.91/0.95, 0.91/0.90; 2F-RV 0.83/0.97, 0.87/0.97, 0.89/0.89. Ungradable rates were lowest for AU (1F: 2.23%, 2F: 1.79% and 5F: 0%) compared with SS (1F: 7.59%, 2F: 4.02% and 5F: 3.56%) and RV (1F: 6.67% and 2F: 5.78%). Table 2 summarises the agreement rates, ungradable rates and SN/SP of 1F, 2F and 5F imaging for DR using the handheld retinal devices. The established SN/SP thresholds (≥80% and ≥95%, respectively) were met by 2F-RV and 5F-AU for any DR, and by 1F-RV, 2F-RV and 5F-AU for refDR. None of the handheld retinal cameras achieved the SN/SP standards for vtDR.

**Table 2:** Comparison of 1F, 2F and 5F fundus imaging using handheld retinal devices

Device/field	Ungradable rate (%)	Severity threshold	K	Kw	Exact agreement (%)	Within one step (%)	SN	SP	PPV	NPV
Aurora										
1F	2.23	Overall	0.26	0.54	44.4	81.3				
		Any DR	0.58				0.69	0.99	0.99	0.61
		refDR	0.62				0.72	0.92	0.89	0.79
		vtDR	0.71				0.80	0.91	0.81	0.90
2F	1.79	Overall	0.31	0.59	48.4	85.8				
		Any DR	0.61				0.72	0.99	0.99	0.63
		refDR	0.66				0.74	0.92	0.88	0.80
		vtDR	0.70				0.81	0.89	0.79	0.91
5F	0	Over-all	0.55	0.75	65.8	93.8				
		Any DR	0.78				0.88	0.97	0.99	0.77
		refDR	0.81				0.86	0.97	0.96	0.87
		vtDR	0.74				0.84	0.92	0.86	0.91
Smartscope										
1F	7.59	Overall	0.27	0.51	42.2	75.6				
		Any DR	0.51				0.72	0.85	0.91	0.59
		refDR	0.65				0.72	0.92	0.89	0.79
		vtDR	0.61				0.67	0.92	0.81	0.85
2F	4.02	Overall	0.35	0.60	49.3	84.0				
		Any DR	0.54				0.76	0.82	0.90	0.63
		refDR	0.67				0.75	0.92	0.89	0.80
		vtDR	0.66				0.75	0.91	0.80	0.88
5F	3.56	Overall	0.50	0.73	60.0	90.7				
		Any DR	0.67				0.83	0.92	0.96	0.69
		refDR	0.79				0.88	0.92	0.92	0.89
		vtDR	0.69				0.87	0.86	0.78	0.92
RetinaVue										
1F	6.67	Overall	0.52	0.77	59.6	90.7				
		Any DR	0.65				0.78	0.93	0.96	0.68
		refDR	0.86				0.91	0.95	0.94	0.92
		vtDR	0.79				0.91	0.90	0.82	0.95
2F	5.78	Overall	0.56	0.75	63.1	88.9				
		Any DR	0.74				0.83	0.97	0.98	0.73
		refDR	0.84				0.87	0.97	0.96	0.89
		vtDR	0.77				0.89	0.89	0.81	0.94

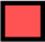
DR thresholds that did not meet the 80% SN or 95% SP rates.

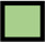
DR thresholds that meet the 80% SN or 95% SP rates.

1F, one-field; 2F, two-field; 5F, five-field; K, kappa value; Kw, weighted kappa; NPV, negative predictive value; PPV, positive predictive value; refDR, referable diabetic retinopathy; SN, sensitivity; SP, specificity; vtDR, vision threatening diabetic retinopathy.

The addition of a second retinal field increased image acquisition time by 30.02±22.66 s (mean±SD) compared with 1F imaging. The addition of three peripheral fields (5F imaging) increased image acquisition time by 90.68±49.95 s compared with 2F imaging and 120.70±63.9 s compared with 1F imaging.

## Discussion

The ever-increasing DR burden in resource-poor settings needs to be addressed innovatively. DR screening with teleophthalmology using handheld retinal imaging devices is one such approach that can broaden the reach of DRSPs in accessing a wider and often hard-to-reach population while increasing the identification of refDR.^13^ Optimising appropriate referrals in imaging-based DRSPs is largely dependent on the imaging protocols deployed. The present study showed that when using handheld retinal imaging devices, the addition of peripheral fields can increase the SN/SP for referable disease and decrease the ungradable rate. The current established minimum standards for SN and SP are 80% and 95% for DR screening.^12 14^ Our results demonstrated that 2F or 5F imaging protocols using some handheld systems for the detection of any DR or refDR can attain these minimum SN/SP thresholds. Moreover, the 5F imaging protocol resulted to an ungradable rate of 0%–3.56%, which is below the UK National Screening Committee recommendation of using imaging devices with ungradable rates of less than 5%.^15^

Previous studies looking at the optimum number of fields used in DR screening showed contrasting results. The majority were published in the 2000s, investigating traditional tabletop cameras, and since then, imaging technology has significantly improved. A 2004 report by the American Academy of Ophthalmology concluded that 1F photography centred on the macula could serve as a sufficient screening protocol for DR, but it was not a substitute for a comprehensive eye exam.^16^ Among level I studies which had the strongest study designs, 1F photography read by certified graders achieved an SN of 61%–90% and SP of 85%–97% compared with ETDRS photos. When compared against ophthalmologists’ clinical examination, SN ranged from 38% to 100%, and SP ranged from 75% to 100%. The wide range of SN/SP with published studies emphasise the need for updated prospective data specifically focusing on newer handheld retinal imaging technology.

Multiple field protocols increase the visible retinal area by providing multiple views of the retina, theoretically leading to improved screening accuracy compared with 1F protocols. Prior studies involving tabletop fundus cameras showed a benefit for increasing the number of fields.^17–20^ In a study by Aptel *et al*, screening accuracy increased from a 1F to 3F protocol.^9^ SN and SP were 0.77/0.99 with a 1F non-mydriatic protocol, 0.92/0.97 with a 3F non-mydriatic protocol, 0.90/0.98 with a 1F mydriatic protocol and 0.97/0.98 with a 3F mydriatic protocol. In another study by Møller *et al*, 1F 60° macula-centred photos missed 11% (4/36) of eyes with retinal neovascularisation found on ETDRS photos.^8^ Furthermore, Vujosevic and colleagues also reported that 1F non-mydriatic 45° macula-centred photography has an SN of only 71% for refDR, lower than the 80% threshold for an effective screening programme, while 3F non-mydriatic 45° photography has an SN of 82%.^21^ They concluded that 1F photography is not suitable for a community-based screening programme and suggested that 3F photography is more applicable for detecting refDR. In contrast, a study by Perrier *et al* comparing a 2F, 3F, and 4F protocol showed a paradoxical decrease in image quality with additional fields.^7^ This was due to difficulty focusing on the additional fields temporal and superotemporal to the macula, which necessitated referrals to the ophthalmologist for poor image quality. Imaging difficulties led to a 6.2% increase in referrals despite the 2F protocol having similar SN/SP as the 3F and 4F protocols, diminishing the cost-effectiveness and utility of the screening approach with more fields. These studies clearly demonstrate the need for updated data to guide current DR screening initiatives with regard to the optimum number of fields. This is especially true for handheld cameras where there is still a need to establish the valid imaging protocols for usage in wide-scale screening programmes.

Handheld retinal imaging devices are portable and relatively inexpensive compact cameras with potential to expand DR screening significantly. These cameras have minimal power consumption, need less space and can be used effectively with less technical training compared with traditional fundus cameras.^22 23^ Barriers to screening including lack of equipment and skilled personnel and financial factors may be partly addressed by validating inexpensive portable technologies that are easy to use for medical imaging and clinical decision making.^24^ However, a review by Cuadros and Bresnick in 2017 concluded that at the time, handheld cameras did not provide adequate image quality for screening, but future design improvements could address their shortcomings.^25^ A study using a smartphone-based non-mydriatic fundus camera has shown promising results.^26^ The SN was 93.1%–94.3% and the SP was 89.1%–94.5% for any DR compared with dilated clinical examination. In another study from India, the SN/SP to detect any DR was 0.75/0.95, while the detection of sight-threatening DR was 0.83/0.99 when using smartphone-based non-mydriatic retinal photography compared with seven-field mydriatic fundus imaging with a tabletop camera.^27^ Our group previously reported that handheld retinal imaging devices attained substantial agreement with mydriatic standard seven-field photography and met the established standards for SN/SP in identifying refDR.^13^ Depending on the camera used, non-mydriatic handheld retinal imaging can achieve sensitivities of 0.87–0.93 and specificities of 0.76–0.92, while mydriatic imaging can achieve sensitivities of 0.84–0.91 and specificities of 0.54–0.97 in identifying refDR.^13^

In the present study, there was increasing agreement with ETDRS photos and increasing SN/SP values noted as the number of fields increased from 1F to 5F when using the AU and SS devices. This trend was not observed when the number of fields increased from 1F to 2F when using the RV device, meaning there was no significant effect when adding just the disc-centred images, highlighting the need for peripheral imaging when using handheld cameras. Compared with 1F imaging, the addition of peripheral fields also decreased ungradable rates by 13%–100% (AU: 100% reduction (1F 2.23%, 5F 0%); SS: 53% reduction (1F 7.6%, 5F 3.6%); and RV: 13% reduction (1F 6.7%, 2F 5.8%)). Given that handheld retinal imaging devices typically have higher ungradable rates and lower agreement rates compared with conventional tabletop cameras, the inclusion of peripheral fields becomes important to add to the imaging protocol for identifying refDR when using portable cameras. From a screening programme’s perspective, reducing the number of fields is helpful as it decreases examination time, increases patient comfort, may enhance compliance to screening schedules, and lowers the number of images that need to be stored and graded per patient.^28^ These must be balanced by possible trade-offs on screening accuracy, especially since DR is largely asymptomatic until the onset of vtDR, and treatment delays could result in suboptimal outcomes. Multiple field protocols may require a more skilled photographer to take fundus photographs since a more refined technique is needed to focus outside the macula and optic nerve while also taking more time to obtain and interpret images. Our results showed that imaging takes approximately 30 s per field; hence, with five fields, the image acquisition time using handheld cameras is around 2.5 min per eye or around 5 min per patient. Although the use of fewer retinal fields is more convenient and less labour intensive, using 5F in screening programmes may be more favourable in detecting early stages of the disease, allowing for prompt referrals and timely treatment. The use of 5F increased image acquisition time on average by 1.5 min compared with 2F and by 2 min compared with 1F. However, this increase in image acquisition time also comes with an increase in both SN and SP as well as a substantial reduction in ungradable rates. In the future, as we move towards automated retinal image analysis for the detection of refDR, this additional grading of peripheral retinal fields may no longer be a significant burden. Furthermore, the additional retinal images allow a greater area of the retina to be visualised and may potentially improve the overall performance of automated systems using artificial intelligence (AI). Various AI models for DR screening are in development, with some already approved by regulatory authorities in some countries.^1 29^ This alternative strategy for DR screening using AI will reduce the reliance on eye care providers and human graders to detect DR, provide methods for triaging high-risk eyes that need urgent medical attention and recognise low-risk eyes that can be safely maintained on image-based follow-up.

To the best of our knowledge, the current study is the first to investigate different numbers of fields when using handheld fundus cameras and their impact on DR screening compared with standard seven-field ETDRS photos. While there have been multiple prior studies investigating the effect of the number of fields on DR severity grading, comparing this study directly with such previous reports using tabletop cameras may not be appropriate due to differences in the imaging technologies between handheld and conventional tabletop cameras. At present, no handheld devices are approved for use in the UK Diabetic Eye Screening Programme, one of the largest and most established DR screening initiatives worldwide.^30^ The data on cheaper alternative but good quality cameras that are more accessible for LMICs need to be expanded, as the burden of vision loss from DR disproportionately affects these regions with poor access to public health services.^10 31^

The strengths of this study include the standardised evaluation of retinal images by certified graders and the use of standardised data collection forms with highly customised electronic medical records designed specifically to evaluate DR outcomes. The gold standard diagnosis was based on grading ETDRS photos, considered a more validated and reproducible method for DR severity grading than a clinical exam. A limitation of this study is that it was conducted at a single hospital with a majority Asian patient population. The effect of pupillary status on the SN/SP values as well as on ungradable rates in the detection of DR when using handheld retinal imaging devices was published in a prior report.^13^ The effect of the imaging protocol when using various handheld cameras for the identification of diabetic macular oedema (DMO) was also reported previously.^13^ Handheld retinal imaging with and without pupil dilation using 2F and 5F has substantial levels of agreement with ETDRS photos for identifying DMO. Furthermore, pupil dilation enhances DMO evaluation by decreasing the ungradable rate allowing attainment of SN thresholds.^13^ Studies comparing 5F handheld imaging with ultrawide field (UWF) images provide further support to our findings of increasing SN/SP with an increasing number of fields captured. Data from UWF and 5F imaging comparisons show that when PDR was the referral threshold for handheld devices, 37.0% of eyes or 30.8% of patients with PDR were missed. Due to the identification of neovascular lesions outside of the handheld fields, lower referral thresholds are needed if handheld devices are used.^32^ Meanwhile, the cost-effectiveness analysis of different imaging protocols when using handheld retinal cameras for DR screening is the subject of a future separate report.

## Conclusion

This study demonstrated that increased number of imaging fields increased the SN and SP for detection of refDR when using handheld retinal imaging devices. With 2F and 5F imaging protocols, the required SN and SP thresholds for clinical use were attained using some, but not all, cameras. These data suggest that handheld retinal imaging performed with additional fields and certain specific systems is accurate enough for DRSPs where their size, cost and ease of use would allow them to be widely deployed in underserved settings. When using a 5F imaging protocol, the ungradable rate can be reduced to a minimum, as low as 0% with some systems, leading to further improvement in patient care.

## Data Availability

Data are available upon reasonable request. All data relevant to the study are included in the article or uploaded as supplementary information. All data analysed during the study are included in this manuscript. Further enquiries can be directed to the corresponding author.
